# Multiple Genetic Modifiers of Bilirubin Metabolism Involvement in Significant Neonatal Hyperbilirubinemia in Patients of Chinese Descent

**DOI:** 10.1371/journal.pone.0132034

**Published:** 2015-07-06

**Authors:** Hui Yang, Qian Wang, Lei Zheng, Min Lin, Xiang-bin Zheng, Fen Lin, Li-Ye Yang

**Affiliations:** 1 Laboratory Medical Center, Nanfang Hospital, Southern Medical University, Guangzhou, Guangdong Province, P. R. China; 2 Central Laboratory, Chaozhou Central Hospital Affiliated to Southern Medical University, Chaozhou, Guangdong Province, P. R. China; NIH, UNITED STATES

## Abstract

The potential for genetic variation to modulate neonatal hyperbilirubinemia risk is increasingly being recognized. A case-control study was designed to assess comprehensive contributions of the multiple genetic modifiers of bilirubin metabolism on significant neonatal hyperbilirubinemia in Chinese descendents. Eleven common mutations and polymorphisms across five bilirubin metabolism genes, namely those encoding UGT1A1, HMOX1, BLVRA, SLCO1B1 and SLCO1B3, were determined using the high resolution melt (HRM) assay or PCR-capillary electrophoresis analysis. A total of 129 hyperbilirubinemic infants and 108 control subjects were evaluated. Breastfeeding and the presence of the minor A allele of rs4148323 (UGTA*6) were correlated with an increased risk of hyperbilirubinemia (OR=2.17, P=0.02 for breastfeeding; OR=9.776, P=0.000 for UGTA*6 homozygote; OR=3.151, P=0.000 for UGTA*6 heterozygote); whereas, increasing gestational age and the presence of –TA_7_ repeat variant of UGT1A1 decreased the risk (OR=0.721, P=0.003 for gestational age; OR=0.313, P=0.002 for heterozygote TA_6_/TA_7_). In addition, the SLCO1B1 and SLCO1B3 polymorphisms also contributed to an increased risk of hyperbilirubinemia. This detailed analysis revealed the impact of multiple genetic modifiers on neonatal hyperbilirubinemia. This may support the use of genetic tests for clinical risk assessment. Furthermore, the established HRM assay can serve as an effective method for large-scale investigation.

## Introduction

Neonatal jaundice or hyperbilirubinemia frequently manifests as a pediatric complex trait or disorder, which is still prevalent (1%) in the newborn population today [1, 2]. Both genetic and environmental factors contribute to the development of neonatal hyperbilirubinemia, and the importance of genetic contributions in this disorder has been recently recognized [3–5].

The main feature of neonatal hyperbilirubinemia is an increased bilirubin production that cannot be matched by glucuronidation and the elimination of bilirubin [6]. Growing literature has shown that genetic variations across multiple bilirubin metabolism genes might affect serum bilirubin levels in healthy adult populations of different ethnic backgrounds [7–11]. However, the role of the genetic modifiers of bilirubin metabolism on neonatal hyperbilirubinemia has not yet been conclusively elucidated.

To explore the complex role of multiple genetic modifiers on unconjugated neonatal hyperbilirubinemia, eleven common polymorphisms across five bilirubin metabolism genes [Heme oxygenase-1 (HMOX1), biliverdin reductase A (BLVRA), hepatic bilirubin-conjugating isoenzyme uridinediphosphoglucuronosyltransferase 1A1 (UGT1A1), and solute carrier organic anion transporter family member 1B1 (SLCO1B1) and 1B3 (SLCO1B3)] were determined in our case-control study of Chinese neonates. In addition, we developed a rapid genotype screening assay for a comprehensive analysis of the nine single base polymorphisms of the eleven selected SNPs using PCR and high-resolution melt analysis (HRM) technology. All selected variants, along with clinical parameters (sex, age and feeding method), were included in our associated evaluation. The data obtained in this study may broaden our knowledge on the molecular pathogenesis of neonatal hyperbilirubinemia and provide genetic markers for clinical risk assessment.

## Materials and Methods

### Study population and sample collection

This was a retrospective case-control study conducted in the pediatric center of a single hospital (Chaozhou Central Hospital afflicted to Southern Medical College). Peripheral blood samples were prospectively collected from newborns consecutively admitted to the study center from November 2011 to September 2014. Clinical records including the birth date, gender, birth weight, delivery method, gestational age, feeding method, total serum bilirubin levels (TSB) and peak bilirubin levels before phototherapy were reviewed. Eligible infants were term infants with a gestational age of more than 37 weeks, a birth weight >2500 g and no major birth abnormalities and serious illness.

Hyperbilirubinemia was diagnosed and treated according to the updated clinical guidelines of the Chinese Medical Association for neonates [12]. The recorded peak TSB was used to divide the study subjects into case and control subjects. The case subjects included jaundiced infants with a maximum TSB that required phototherapy based on the above guidelines. Neonates with known clinical risk factors for developing neonatal hyperbilirubinemia, such as hemolysis (a positive Coombs’ test), glucose-6-phosphate dehydrogenase deficiency, cephalohematoma, infection, perinatal asphyxia, and major organ abnormality, were excluded. Control subjects were term neonates, admitted to the study center for other reasons than jaundice during the same period, with a TSB not requiring phototherapy according to the same guidelines.

The study was approved by Ethics Committee of Chaozhou Central Hospital. Because the data were analyzed anonymously and blood samples for this study were used after the completion of clinical diagnostic work (blood routine examination), the hospital ethics committees approved a waiver of written consent.

### Molecular Analysis

The genomic DNA was extracted from surplus EDTA anti-coagulated whole blood samples using a DNA mini-preparation kit (Decipher Bioscience Shenzhen Ltd., Shenzhen, China). 100uL anti-coagulated whole blood was used for each DNA specimen extraction. The specific mutations of UGT1A1, HO-1, BLVRA, SLCO1B1, and SLCO1B3 were analyzed as described below. The information on the eleven polymorphisms was listed in S1 Table.

The (GT)_n_ repeat variations in the HMOX1 (dbSNP rs1805173) and the (TA)_n_ repeat variations in the UGT1A1(dbSNP rs81753472) gene promoters were determined by fragment (size-based) analysis. Both promoter regions were separately amplified by polymerase chain reaction (PCR) with a FAM-labeled sense primer. The PCR products were mixed with the Gene Scan-400 size standard (size range: 50–400 bp, Applied Biosystems Inc.), and analyzed on the ABI 3700 Genetic Analyzer (Applied BioSystems, Foster City, California) using the software GeneMapper 4.0 (Applied BioSystems). To confirm the sizes of the (GT)_n_ and (TA)_n_ repeats, selected samples were subjected to sequence analysis on an ABI Genetic Analyzer. The sequence results were used to confirm the molecular weight as determined by fluorescence labeling.

The polymorphisms rs4148323, rs35390960, rs6742078 and rs1018124 from the UGT1A1 gene, rs699512 from the BLVRA gene, rs2306283 and rs4149056 from the SLCO1B1 gene, and rs2417940 and rs2117032 from the SLCO1B3 gene were identified by PCR and HRM analysis using Lightcycler instruments. The reaction conditions were 95°C for 3 min, followed by 10 cycles of 98°C for 10 s, 65°C for 5 s, and 72°C for 15 s, and 50 cycles of 98°C for 10 s, 55°C for 5 s, and 72°C for 15 s, with final extension at 72°C for 90 s. The cycling conditions were the same for all amplicons. After amplification, HRM analysis was performed using the software LightCycler 480 SW 1.5 (Roche Diagnostics) as described previously [13, 14]. Each DNA sample was amplified twice to validate the coherence of the curve shapes. Selected samples were amplified and directly sequenced to verify the accurate genotype of the HRM assay. Primers for the HRM assay were given in S1 Table.

### Statistical analysis

χ2-test, Fisher’s exact test, Student’s t-test, and the Mann-Whitney nonparametric test were used to compare the differences in the clinical parameters, as appropriate. The Hardy-Weinberg equilibrium (HWE) test for the (GT)_n_ repeat of HMOX1 was performed using the exact test [15]. The HWE test for the other loci was examined using a chi-square test. Linkage disequilibrium (LD) between the 4 polymorphisms within UGT1A1 was calculated, and the analysis of inferred haplotypes was also performed using the web tool SNPStats [16] (http://bioinfo.iconcologia.net/SNPStats). Furthermore, the best model of inheritance for each SNP (co-dominant, dominant, recessive, overdominant or additive) was selected based on the lowest Akaike information criterion.

Logistic regression models were performed to evaluate the association between the specific polymorphism or haplotypes and the development (case vs. control) and severity of neonatal hyperbilirubinemia (severe, mild/moderate jaundice or no jaundice). Initially, the estimated odds ratio (OR) and corresponding 95% confidence interval (CI) were measured between the case and control groups using only binary logistic regression after adjusting for known clinical risk factors for neonatal hyperbilirubinemia, including sex, breastfeeding, and age. Then, a multivariate ordinal logistic regression model was used to evaluate the association of the gene variations and clinical parameters with the severity of hyperbilirubinemia categorized by the peak TSB levels recorded before phototherapy. The threshold for this categorization was taken from the recommendations of the 2003 National Institute of Child Health and Human Development (NIHCD), National Institutes of Health, United States of America conference [1], including serious hyperbilirubinemia (TSB levels ≥20 mg/dL), mild/moderate jaundice (TSB levels ≥12 mg/dL) and no jaundice. The stepwise selection procedure was used to investigate the most significant predictors of hyperbilirubinemia. Variables with P < 0.10 from the separated binary logistic analysis were included in the stepwise selection. Fitting model in the ordinal regression was evaluated by the parallel line test and goodness of fitting test. Both the non-significant P-value (p>0.05) of the parallel line test and goodness of fit test indicated that the ordered models were appropriate [17]. In all the regression analyses, the common homozygote genotype in the control population was considered as the reference category. P<0.1 was considered to be significant. Subset analysis was further carried out to evaluate for a potential synergistic effect between genetic modifiers and covariates, including the sex, breastfeeding status and gestational age of the included neonates.

All statistical analysis was performed using SPSS version 16 for windows (SPSS, Chicago, Illinois, USA) and SNPstat Software. All statistical tests were 2-sided, and statistical significance was set at P<0.05.

## Results

### Clinical characteristics

Eighty-one jaundiced neonates were excluded based on the criteria for exclusion as described previously, and a total of 129 term newborns with significant hyperbilirubinemia, including 42 neonates with peak TSB>20 mg/dL (342 μmol/L), were enrolled as case subjects. All the neonates in the case group received phototherapy except 2 neonates with prolonged unconjugated hyperbilirubinemia. The control subjects were 108 term neonates with no clinical jaundice [median TSB: 97.11 μmol/L (13.1–225.4 μmol/L)]. The clinical features of the subjects were summarized in Table 1. There were no statistically significant differences between case subjects and control subjects with respect to birth weight and sex. Although late preterm neonates were excluded, the gestational age still differed between the two groups with 39.0 ± 1.3 wk vs. 39.6 ± 1.1 wk (P < 0.001). Furthermore, the case neonates were breastfed more often than the control neonates.

**Table 1:** Demographic and clinical features of the neonates in the case and control groups.

	*Case*	*Control*	*p*
Sex (n)			
Male	80	64	0.707
Female	49	44	
Gestational age (week) ^a^	39.0±1.31	39.5±1.20	0.003
Birth weight (kg) ^a^	3.15±0.38	3.16±0.37	0.771
Maximum TSB levels (μmol/L) ^b^	312.48(250.5–564.8)	97.11(13.1–225.4)	
Feeding			0.048
Breastfeeding	59	41	
Breast and formula	40	28	
Formula	24	31	
Unknown	6	8	0.370
Birth delivery			0.104
Vaginal	64	65	
Cesarean	65	43	

^a^ Mean±SD.

^b^ Median (95% CI).

### Genotype results

**Fig 1:**
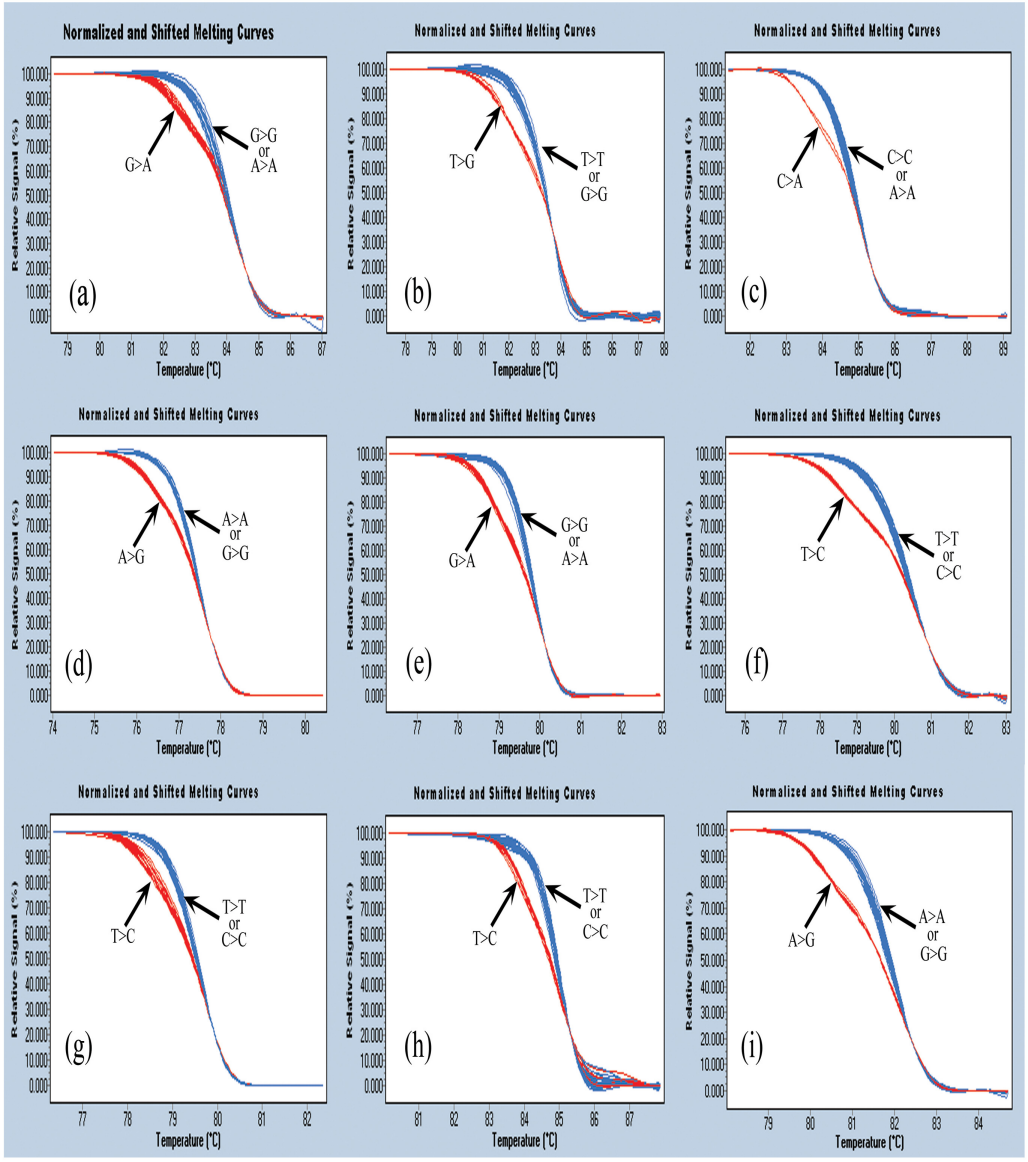
HRM analysis of 9 single base polymorphisms across the UGT1A1, SLCO1B1, SLCO1B3 and BLVRA genes. (a): rs4148323 G>A; (b): rs6742078 T>G; (c):rs35390940 C>A; (d): rs108124 A>G; (e): rs2306283 G>A; (f): rs4149056 T>C; (g): rs2117032 T>C; (h): rs2417940 T>C; (i): rs699512 A>G.

HRM analysis was applied for the rapid genotyping of the 9 selected common single base variants in our study cohort. As shown in Fig 1, a heterozygous mutation could be easily distinguished from wild-type samples based on differences in the HRM curve shape. The HRM curve shapes for the homozygous mutations were similar to those of the wild-type subjects, and the homozygous mutations were detected by a modified HRM analytical strategy as described in our previous study [13]. Each SNP for the 50 samples was selected for direct sequencing. All the test samples were accurately genotyped as confirmed by the sequencing results. Therefore, we believed that HRM could be used as a general and rapid method for large-scale clinical investigation.

The minor allele frequencies (MAFs) of the polymorphisms in our study cohort were presented in Table 2. The MAF of SNP rs3539046 in UGT1A1 was lower than 0.003 and was excluded from further analysis. None of the polymorphisms showed statistically significant deviations from the HWE in our control subjects except rs4149056, which was excluded from the sub-analysis. Strong pairwise LD was observed between the five polymorphisms (each |D|’>0.8) within UGT1A1, whereas a low pairwise LD was present between the polymorphisms within the SLCOs family (SLCO1B1, SLCO1B3). The LD pattern across the multiple SNPs of UGT1A1 was shown in S2 Table. A high r^2^ value (0.885) was present only between the (TA)_n_ repeat polymorphism and the rs6742078 of UGT1A1 gene.

**Table 2:** Minor allelic, genotypic, and haplotype distributions of the 11 polymorphisms in the bilirubin metabolism gene: control vs. case groups.

polymorphism	location	control	case	*P*_H-W_ ^c^	*P*_allele_	*P*_genotype_
HO-1^a^						
(GT)_n_	promoter			0.34	0.38	0.59
S allele		54 (25.2%)	80 (31.0%)			
M allele		56 (26.2%)	63 (24.4%)			
L allele		104 (48.6%)	115(44.6%)			
SS		7 (6.5%)	13 (10.1%)			
SM		10 (9.3%)	17 (13.2%)			
SL		30 (28.0%)	37 (28.7%)			
MM		7 (6.5%)	4 (3.1%)			
ML		32 (29.9%)	38 (29.5)			
LL		21 (19.6%)	20 (15.5%)			
UGT1A1						
(TA)_n_	promoter			0.052	0.0002	8.38e-05
TA_7_		34 (15.7%)	14 (5.4%)			
TA_6_		182 (84.3%)	244(94.6%)			
TA_7_/TA_7_		0 (0.00%)	0 (0.00%)			
TA_6_/TA_7_		34 (31.5%)	14 (10.9%)			
TA_6_/TA_6_		74 (68.5%)	115(81.9%)			
rs4148323	Exon 1					
A allele		25 (11.7%)	74 (28.7%)	0.666	6.39e-06	7.06e-05
A/A		1 (0.9%)	12 (9.3%)			
G/A		23 (21.5%)	50 (38.8%)			
G/G		83 (77.6%)	67 (51.9%)			
rs35390960	Exon 1			0.78	0.13	0.13
A allele		5 (2.7%)	2 (0.8%)			
A/A		0 (0.00%)	0 (0.00%)			
C/A		5 (5.5%)	2 (1.7%)			
C/C		86 (94.5%)	116(98.3%)			
rs6742078	Intron 1			0.059	1.64e-05	5.52e-06
G allele		33 (15.4%)	9 (3.9%)			
G/G		0 (0.00%)	0 (0.00%)			
T/G		33 (30.8%)	9 (7.8%)			
T/T		75 (69.2%)	117(92.2%)			
rs108124	Intron 1			0.37	0.31	0.54
G allele		36 (18.4%)	53 (22.3%)			
G/G		2 (2.0%)	3 (2.5%)			
A/G		32 (32.7%)	47 (39.5%)			
A/A		64 (65.3%)	69 (58%)			
SLCO1B1						
rs2306283	Exon 5			0.428	0.11	0.22
A allele		49 (22.7%)	75 (29.1%)			
A/A		7 (6.5%)	17 (13.2%)			
G/A		35 (32.4%)	41 (31.8%)			
G/G		66 (61.1%)	71 (55.0%)			
rs4149056	Exon 6			0.003	-	-
C allele		39 (18.2%)	33 (12.8%)			
C/C		8 (7.5%)	2 (1.6%)			
T/C		23 (21.5%)	29 (22.5%)			
T/T		76 (71.0%)	98 (76.0%)			
SLCO1B3						
rs2117032	3’-UTR			0.50	0.98	0.23
C allele		101 (48.1%)	121(48.0%)			
C/C		26 (24.8%)	24 (19.0%)			
T/C		49 (46.7%)	73 (57.9%)			
T/T		30 (28.6%)	29 (23.0%)			
rs2417940	Intron 7			0.65	0.93	0.91
T allele		37 (18.0%)	46 (18.3%)			
T/T		4 (3.9%)	4 (3.2%)			
C/T		29 (28.2%)	38 (30.2%)			
C/C		71 (68%)	84 (66.7%)			
BLVRA						
rs699512	Exon 2			0.48	0.46	0.93
G		56 (26.4%)	76 (29.5%)			
G/G		6 (5.7%)	11 (8.5%)			
A/G		44 (41.5%)	54 (41.9%)			
A/A		56 (52.6%)	64 (49.6%)			
Haplotype^b^						
ATA6		72 (30.5%)	22 (11.7%)			3.64e-06
GTG6		53 (22.4%)	34 (17.4)			0.21
GGA7		9 (3.8%)	31 (16.1%)			1.21e-05
GTA7		4 (1.7%)	1 (0.5%)			0.34
GTA6		98 (41.6%)	103(53.4%)			0.012
Others		-	-			

^a^ S≤23(GT)_n_; 23<M≤29(GT)_n_; L>29(GT)_n_.

^b^ other haplotypes had frequencies less than 1%.

^c^ Hardy-Weinberg Equilibrium test *p* value.

The number of (GT)_n_ repeats in the HO-1 gene promoter region in this study population ranged from 15 to 40, with one peak at 23 GT repeats and the other peaks at close to 30 GT repeats. According to previous report from the HO-1 association and functional studies [18, 19], we divided the allelic repeats into three subgroups: short alleles (S: <24 GT), middle alleles (M: 24–29 GT), and long alleles (L: >29 GT). We then further classified the 6 genotypes into 2 groups as was done in previous studies [7, 20]. Subjects carrying the S/S or S/M GT repeats were classified as Group 1, whereas subjects carrying M/M, M/L, or L/L GT repeats were Group 2.

### Association study

Association analysis of the 10 common variants with hyperbilirubinemia was first evaluated using separate binary logistic regressions (SNPstas software). The allele, genotype and haplotype of the three SNPs in UGT1A1, including rs4148323, rs6742078 and (TA)_n_, were found to have considerable differences between cases and controls. There was also a strong association with neonatal hyperbilirubinemia after being adjusted for known clinical risk factors for neonatal hyperbilirubinemia, including sex, breastfeeding, and age (Tables 2 and 3). Specifically, neonates harboring minor alleles of rs4148323 (known as UGT1A1*6, 211G>A) were found to have a significantly increased risk of hyperbilirubinemia (OR_adj_ = 13.02; p = 1e-04 for UGT1A1*6 homozygote, OR_adj_ = 2.69; p = 1e-04 for UGT1A1*6 heterozygote), whereas minor alleles at rs6742807 and (TA)_n_[rs8175347] were observed to have a protective effect on the risk of hyperbilirubinemia (OR_adj_ = 0.16; p = 0.0001 for rs6742807; OR_adj_ = 0.25; p = 0.000 for (TA)_n_). Similarly, haplotype analysis showed that ATA(TA)_6_ (rs4148323-rs6742078-rs108124-(TA)_n_) increased the risk (OR_adj_ = 3.00; p = 4e-04), whereas GAT(TA)_7_ produced a protective effect for hyperbilirubinemia (OR_adj_ = 0.22; p = 0.0018) compared to the most common haplotype GTA(TA)_6_. Two SNPs in the SLCOs family also showed a trend towards an increased risk of hyperbilirubinemia in the univariable regression analysis after adjusting for potential covariates, including the age, gender, and feeding method (OR_adj_ = 2.16; p = 0.098 for rs2306283 in recessive model; OR_adj_ = 1.61; p = 0.096 for rs2117032 in overdominant model).

**Table 3:** Association analysis of the 10 polymorphisms in bilirubin metabolism genes and the risk of hyperbilirubinemia under different inheritance models: binary logistic regression.

	OR_crude_(95%CI)	*P*_crude_	OR_adj_ ^a^(95%CI)	*P*_adj_ ^a^
Ho-1				
(GT)_n_				
SS+SM	1.60 (0.83–3.10)	0.16	1.70 (0.83–3.48)	0.14
SL+MM+ML+LL	reference			
UGT1A1				
(TA)_n_				
TA_6_/TA_7_	0.26 (0.13–0.53)	1e-04	0.25 (0.12–0.50)	1e-04
TA_6_/TA_6_	reference			
rs4148323				
A/A	14.87(1.89–117.08)	<0.0001	13.02(1.61–105.53)	1e-04
G/A	2.69 (1.49–4.86)	<0.0001	2.69 (1.43–5.06)	1e-04
G/G	reference			
rs6742078				
T/G	0.19 (0.09–0.41)	<0.0001	0.16 (0.07–0.36)	<0.0001
T/T	reference			
rs108124				
A/G+G/G	1.36 (0.78–2.37)	0.27	1.41 (0.78–2.56)	0.25
A/A	reference			
BLVRA				
rs699512				
G/G	1.55 (0.55–4.35)	0.39	1.99 (0.63–6.25)	0.23
A/A+A/G	reference			
SLCO1B1				
rs2306283				
A/A	2.19 (0.87–5.50)	0.083	2.16 (0.84–5.53)	0.098
G/G+G/A	reference			
SLCO1B3				
rs2117032				
T/C	1.57 (0.93–2.65)	0.087	1.61 (0.92–2.82)	0.096
T/T+C/C	reference			
rs2417940				
C/T	1.10 (0.62–1.96)	0.74	1.06 (0.58–1.94)	0.84
C/C+T/T	reference			
Haplotype ^b^ (Frequency)				
ATA6 (20.64%)	3.17 (1.80–5.58)	1e-04	3.00 (1.66–5.43)	4e-04
GTG6 (20.63%)	1.42 (0.78–2.58)	0.25	1.36 (0.73–2.53)	0.33
GGA7 (9.07%)	0.26 (0.12–0.60)	0.0018	0.22 (0.09–0.52)	7e-04
GTA7 (1.05%)	4.24 (0.46–39.46)	0.21	6.67 (0.67–66.00)	0.11
GTA6 (48.28%)	reference			
Others^c^ (0.31%)	-	-	-	-

^a^ Adjusted for age, gender, and feeding practice.

^b^ polymorphisms are in order of: rs4148323-rs6742078-rs108124-(TA)_n_.

^c^ other haplotypes had frequencies of less than 1%.

The neonates were further stratified into three groups by the peak TSB levels recorded for each neonate with severe (TSB levels ≥20 mg/dL) or mild/moderate hyperbilirubinemia (TSB levels ≥12 mg/dL) or no jaundice (as recommended by 2003 NIHCD conference) [1]. In the multivariate ordinal regression analysis, the following 4 SNPs, together with 2 demographic predictors were significant and remained in the model. The estimated OR and 95%CI of the six predictors of hyperbilirubinemia were shown in Table 4. Neonates of older gestational age and TA_7_ repeat variants of UGT1A1 (UGTA*28) decreased the risk of hyperbilirubinemia (OR = 0.721, 95%CI: 0.583–0.895, P = 0.003 for gestation age; OR = 0.313, 95%CI: 0.148–0.660, P = 0.002 for heterozygote TA_6_/TA_7_), whereas breastfeeding and presence of minor A allele of rs4148323 (UGTA*6) increased the hyperbilirubinemia risk (OR = 2.17, 95%CI: 1.127–4.203, P = 0.02 for breastfeeding; OR = 9.776, 95%CI: 2.812–34.02, P = 0.000 for UGTA*6 homozygote; OR = 3.151, 95%CI: 1.729–5.748, P = 0.000 for UGTA*6 heterozygote). In addition, the homozygote A/A of rs2306283 in SLCO1B1 and the heterozygote C/T of rs2117032 in SLCO1B3 also contributed to an increased risk of hyperbilirubinemia (OR_adj_ = 2.401, 1.037–5.556, p = 0.041 for rs2306283 in the recessive model; OR_adj_ = 2.10, 1.198–3.684, p = 0.01 for rs2117032 in the overdominant model).

**Table 4:** Ordinal logistic model: association of genetic and clinical risk factors with the severity of hyperbilirubinemia according to the TSB level.

Variable	Parameter estimation				Model fitting information (χ^2^ statistic)*			
	Wald χ^2^ statistic	*P* value	Odd ratio	95%CI		χ^2^ statistic	*P* value	R^2^
GW	8.758	0.003	0.721	0.583~0.895	Location	F = 61.49	0.000	C = 0.248
Feeding way					Model	P = 175.78	0.201	N = 0.283
Feeding(1) ^a^	5.375	0.020	2.17	1.127~4.203		D = 158.98	0.53	M = 0.137
Feeding(2)	reference					L = 2.572	0.922	
rs4148323					Scale	F = 67.36	0.000	C = 0.268
A/A	12.858	3.35e-4	9.776	2.812~34.021	Model	P = 161.62	0.321	N = 0.306
G/A	14.048	1.79e-4	3.151	1.729~5.748		D = 153.11	0.505	M = 0.150
G/G	reference							
(TA)_n_								
TA_6_/TA_7_	9.312	0.002	0.313	0.148~0.660				
TA_6_/TA_6_	reference							
rs2306283								
A/A	4.187	0.041	2.401	1.037~5.556				
G/G+G/A	reference							
rs2117032								
T/C	6.707	0.010	2.100	1.198~3.684				
T/T+C/C	reference							

*Abbreviation: GW: Gestational week; F: Model fitting statistic; P: Pearson χ^2^ statistic; D: Deviance χ^2^ statistic; L: Parallel line test statistic. C: Cox and Snell; N: Nagelkerke; M: McFadden. The parallel line test was tested using the χ^2^ statistic. A non-significant P value indicated that the odds ratio could be interpreted as constant across all possible cut-off points of the outcome (Null hypothesis). This Null hypothesis was applied for only the evaluation of the location model. Pearson χ^2^ and Deviance χ^2^ were used to test the Goodness of fitting. If the P value of the χ^2^ statistic was less than 0.05, we rejected the null hypothesis and concluded that there was a significant difference between the observed and expected values. The larger the R^2^, the better the model fit.

^a^ The feeding (1) group was comprised of breast or mixed breast and formula fed infants; the feeding (2) group was comprised of exclusively formula fed infants.

In the subgroup analysis, the neonates were stratified by age, sex and feeding method, and we found that there existed an additive effect of the UGTA*6 variant (211 G >A, Gly71Arg) and breastfeeding on the hyperbilirubinemia risk. As shown in Table 5, after adjusting for the sex and gestational age, the neonates carrying heterozygous and homozygous UGTA*6 variants (G/A and A/A genotype) had a substantially higher risk of hyperbilirubinemia than those with the wild phenotype (G/G genotype) in the BF group (breastfed and mixed breastfed) rather than in the SF group (exclusively supplement formula-fed). Neonates with the homozygous UGTA*6 variant that were exclusively fed with breast milk had the highest relative risk of hyperbilirubinemia development (*P* = 0.018).

**Table 5:** Subgroup analysis of rs4148323 polymorphisms and the risk of hyperbilirubinemia depending on the feeding type.

*Feeding type*	*rs4148323*											
	*Wildtype*			*G/A*			*A/A*			*G/A+A/A*		
	Control (n)	Case (n)	OR_adj_ * (95%CI)	Control (n)	Case (n)	OR_adj_ * (95%CI)	Control (n)	Case (n)	OR_adj_ * (95%CI)	Control (n)	Case (n)	OR_adj_ * (95%CI)
Formula-fed	19	18	reference	9	4	0.47(0.12–1.85)	1	2	2.00(0.16–25.38)	10	6	0.63(0.18–2.14)
Breast and formula	23	21	0.95(0.39–2.32)	**5**	**15**	**3.60(1.05–12.35)**	**0**	**4**	**---**	**5**	**19**	**4.55(1.36–15.22)**
Breastfed	34	27	0.76(0.33–1.75)	**6**	**27**	**4.57(1.50–13.90)**	**0**	**5**	---	**6**	**32**	**5.33(1.77–16.10)**

*Adjusted for sex and gestational age.

^#^ Subgroups with P<0.05 are shown in bold.

## Discussion

The pathogenesis of significant neonatal hyperbilirubinemia is often multifactorial, involving bilirubin overproduction, reduced conjugation and increased enterohepatic recycling [6, 21]. The purpose of the present study was to elucidate the comprehensive contributions of the multiple genetic modifiers of bilirubin metabolism on the development of significant hyperbilirubinemia in newborns of Chinese descent. Our results revealed that two independent genetic variants in the promoter and coding region of UGT1A1 genes had a substantial impact on the risk of neonatal hyperbilirubinemia. In addition, two polymorphisms in the SLCO family were also associated with hyperbiliruinemia risk in our study cohort. Finally, our data demonstrated that there was a significant gene-environment interaction between the UGT1A1 gene coding region variation and breastfeeding.

The UGT1A1 coding sequence variant rs4148323 (known as UGT1A1*6, G211A), the most common cause of Gilbert syndrome in east Asians, was well documented and predominantly associated with TSB levels and neonatal hyperbilirubinemia risk in the Asian population [22–24]. Our results confirmed the strong association of UGT1A1*6 with the incidence and severity of hyperbilirubinemia. Furthermore, we observed a significant gene-environment interaction between UGT1A1 *6 and breastfeeding. Recently, Chou et al. in Taiwan found that UGT1A1*6 was significantly associated with hyperbilirubinemia in exclusively breastfed neonates. In the current study, we further demonstrated that UGT1A1*6 was also a risk factor in mixed breastfed neonates. Moreover, we clearly demonstrated that UGT1A1*6 was not a significant risk predictor in neonates who were exclusively formula-fed. This was consistent with Chou’s observations [25], but was somewhat inconsistent with the results of a similar study in Taiwanese neonates by Huang et al. [22], who showed that UGT1A1 *6 was also a risk factor of neonatal hyperbilirubinemia in neonates who were not fed with breast milk. The discrepancy between these findings may be due to differences in the categorization of the study populations. The supplement formula-fed group in our study included those neonates who were exclusively formula-fed. In contrast, the neonates in Chou and in Huang’s study received both formula and breast milk.

Several mechanisms have been proposed to explain the additive role of UGT1A1 gene variations and breastfeeding in significant neonatal hyperbilirubinemia. It has been demonstrated that pregnane-3(a), 20(b)-diol in breast milk inhibited bilirubin conjugation in the presence of the UGT1A1*6 polymorphic mutation of UGT1A1 [26]. Furthermore, later studies using the humanized UGT1 mouse model have clarified that breast milk reduced the expression of intestinal UGT1A1, which enhanced the risk of hyperbilirubinemia because UGT1A1 expression in the small intestine played an important role in bilirubin glucuronidation during the neonatal period [27, 28]. Although the UGT1A1*6 genotype was not evaluated in the UGT1A1*1 and UGT1A1*28 mouse models, these mechanisms explained the additive role of UGT1A1 gene variations and breastfeeding in significant neonatal hyperbilirubinemia.

The (TA)_n_ repeat variant in the UGT1A1 promoter was another extensively studied variant. Previous epidemiological studies based on independent samples or GWAs samples from European and Asia adult populations have demonstrated that the long repeat of TA_n_ was associated with increased TSB levels [7, 8, 10, 11, 29]. However, the role of this variant on neonatal hyperbilirubinemia risk was undefined and conflicting. For instance, homozygous A(TA)_7_TAA variations in the promoter region of the UGT1A1 gene was found to be associated with neonatal hyperbilirubinemia in Caucasian, whereas most studies in east Asian countries failed to find this association [30]. Interestingly, two recent case-control studies in Chinese and Japanese breastfed neonates observed that the heterozygous (TA)_7_ mutation decreased the risk of hyperbilirubinemia significantly (OR: 0.37; 95%CI: 0.15–0.89; p = 0.027) [20, 31]. In the present study, we also found an inverse association between the TA_7_ repeat variant and the hyperbilirubinemia risk. Taken together, the TA_7_ repeat variant of UGT1A1 (UGTA*28) seems to have a protective effect on hyperbilirubinemia development in Asia neonates. Although this conclusion contradicted the results of the traditional function study, Zhou el al further found higher bilirubin levels in neonates heterozygous for (TA)_7_/(TA)_6_ than in those homozygous for (TA)_7_/(TA)_7_ [32]. One study in Taiwanese neonates also reported a dose-dependent effect of UGTA*28 on the lower TSB levels (homozygote<heterozygote<wild type) [25]. Another study in jaundiced children also observed this trend, although the ethnicity of the subjects was not described [33]. It was unusual that the promoter variant showed different, even opposite, effects on TSB. The first suggested mechanism for this phenomenon come from Beutler et al. [34], who suggested that the (TA)_n_ repeat might be a balanced polymorphism evolutionarily selected to maintain serum bilirubin in an optimal range in the face of largely undefined genetic and environmental pressures. Further studies are certainly needed to confirm this hypothesis.

Another SNP (rs6742078) in the UGT1A1 gene also showed a protective effect for neonatal hyperbilirubinemia. This SNP was reported to be highly linked with another promoter: SNP rs887829 (r^2^>0.96) [9]. Interestingly, both SNPs were strongly linked with the functional TA_n_ promoter polymorphism (r^2^ = 0.88) and were significantly associated with TSB levels in Asian and European GWAS studies [8, 10]. Therefore, the protective effects of rs6742078 may be attributed to the strong LD between this site and the (TA)_n_ repeat.

SLCO1B1 and SLCO1B3 genes are members of the OATP family, which is highly expressed in the basolateral membrane of hepatocytes. Among the other 6 SNPs in the other 4 bilirubin metabolism genes, only rs2306283 in SLCO1B1 and rs2117032 in SLCO1B3 showed a suggestive association with neonatal hyperbilirubinemia risk. Our finding was in accordance with recent case-control studies in Brazilian and Chinese neonates [35, 36], although two previous GWASs reported discordant results for the two loci of SLCOs on the TSB levels between Korean- and European-derived populations [8, 10]. In addition, the high MAFof rs2306283 and rs2117032 in the current cohort suggested that variants in SLCOs alone might not account for the substantial increased hyperbilirubinemia risk. In another words, there may be an additive effect of SLCO variants and other icterogenic conditions. Together, they were correlated with a significantly increased risk hyperbilirubinemia. This hypothesis was partly supported by Huang et al.’s observation in Taiwan neonates, who reported a significant additive effect of UGT1A1*6 variant and OATP1B1*1b on neonatal hyperbilirubinemia.

Heme oxygenase-1 (HO-1; OMIM*141250) is the initial and rate-limiting enzyme in the conversion of heme to bilirubin. It is believed that a short (GT)_n_ repeat might be associated with a higher TSB levels and thus influence the hyperbilirubinemia risk in newborns [18]. This hypothesis is supported by two case-control studies [37, 38]. Both studies illustrated a significantly increased risk for the development of hyperbilirubinemia in neonates carrying short alleles compared to those carrying longer alleles. However, we did not find any relationship between the (GT)_n_ repeat polymorphism and the hyperbilirubinemia risk in our study cohort, even when we tried different cut-off values to define the ‘short’ and ‘long’ allele classes of the (GT)_n_ repeats. This was consistent with sequential studies by Zhou et al. [20, 32] in Chinese neonates, and Sato et al. on severely jaundiced Japanese neonates [31] The latter group also reported a lack of an association between the short (GT)_n_ repeat variant and an increased risk of neonatal hyperbilirubinemia. Furthermore, Kaplan et al in a population of Israeli neonates showed that (GT)_n_ repeat length did not modulate bilirubin metabolism and TSB level of neonates at the 3 postnatal day [39]. We did not have enough information to explain these discrepant findings. However, these results may suggest that HO-1 is not the major gene involved in the pathogenesis of neonatal hyperbilirubinemia. Therefore, the effects of HMOX1 on the TSB levels could easily be affected by different genetic backgrounds [7] and other icterogenic conditions [40].

We acknowledge that this study has some limitations. The retrospective sampling employed here, which shaped the clinical distribution of the cohort and the sample size, may have limited our ability to definitively identify common variants with relatively small effect sizes of the expected type. For instance, we did not find strong evidence for the role of common variation in the SLCO family on neonatal hyperbilirubinemia risk as we initially evaluated the genetic effect based on the absolute occurrence of significant hyperbilirubinemia. Ideally, when we further divided the study neonates by the severity of their jaundice, the significant effects of the two risk predictors could have been observed. Indeed, this partly suggests that a quantitative approach could significantly increase the statistical power for the detection of genetic factors [41]. The validity of categorizing the study neonates into the 3 above mentioned groups could be questioned. The model fitting information shown in Table 4 showed that the ordered models were appropriate.

Another limitation was that the SNPs employed in the present study did not encompass all common variations at UGT1A1, HO-1, BLVRA, SLCO1B1, and SLCO1B3. However, the present study was economical and included almost all important and known functional common variants in the five bilirubin metabolism genes reported in the Asian population. This would provide a framework for the larger and more comprehensive evaluation of susceptibility genes.

Severe neonatal hyperbilirubinemia is a complex pediatric disorder. The usual studied clinical factors alone cannot provide the real cause. Genetic variance is responsible for this condition. Future study will further clarify the interactions among multiple bilirubin metabolism gene loci, other genes, and nongenetic factors to neonatal hyperbilirubinemia.

In conclusion, this detailed analysis revealed the impact of multiple genetic modifiers on neonatal hyperbilirubinemia, reflecting the complex nature of neonatal hyperbilirubinemia. The data obtained in this study has notably increased our knowledge on the molecular pathogenesis of neonatal hyperbilirubinemia and has provided genetic markers for clinical risk assessment. Furthermore, the established HRM for genotyping common mutations and polymorphisms could be used as a general and rapid method for future large-scale investigation.

## Supporting Information

S1 Table Primers used for genotyping the 11 common polymorphisms across the five bilirubin metabolism genes. (DOC)

S2 Table Linkage disequilibrium (LD) analysis of the *UGT1A1* gene. (DOC)

S3 Table Detailed clinical information of control neonates described in manuscript. (XLS)
