# Supplementary material for: Multiple Genetic Modifiers of Bilirubin Metabolism Involvement in Significant Neonatal Hyperbilirubinemia in Patients of Chinese Descent
Source: PLoS One. 2015 Jul 6;10(7):e0132034. doi: 10.1371/journal.pone.0132034 (PMC4493094; doi:10.1371/journal.pone.0132034)
Supplement: S1 Table — (DOC) [file pone.0132034.s001.doc]

Table S1 Primers used for genotyping the 11 common polymorphisms across the five bilirubin metabolism genes

| Gene | polymorphism | chromosome | Variant position | assay | Primers sequence(5’-3’) |
| --- | --- | --- | --- | --- | --- |
| Ho-1 | (GT)_n_ | 22 | promoter | Genescan | GT-F: FAM-AGCCTGCAGCTTCTCAGATTTC |
|  |  |  |  |  | GT-R: GGGTGGAGAGGAGCAGTCAT |
| UGT1A1 | (TA)_n_ | 2 | promoter | Genescan | F: FAM-ACGTGACACAGTCAAACATTAACT |
|  |  |  |  |  | R: CCAGCATGGGACACCACTG |
|  | rs4148323 | 2 | Exon 1 | HRM | U1F:CACCTGACGCCTCGTTGTAC |
|  |  |  |  |  | U1R: CTCTTTCACATCCTCCCTTTG |
|  | rs35390960 | 2 | Exon 1 | HRM | U2F: ATGCTCATTGCCTTTTCACA |
|  |  |  |  |  | U2R: GGTCCTGGACAGTCACCTCT |
|  | rs6742078 | 2 | Intron 1 | HRM | U3F: GGAGCAGGGAAGGTTGAGAT |
|  |  |  |  |  | U3R: GCACTTAGACACCATGTGGG |
|  | rs108124 | 2 | Intron 1 | HRM | U4F: GATCCACCACACTCAGAAGT |
|  |  |  |  |  | U4R:TCAGTGTTCTAACCAACAGCTT |
| SLCO1B1 | rs2306283 | 12 | Exon 5 | HRM | S1F: CTCTGTATTTCTAGGAAAAGTG |
|  |  |  |  |  | S1R: TAAACAAGTGGATAAGGTCG |
|  | rs4149056 | 12 | Exon 6 | HRM | S2F: ACATAGGTTGTTTAAAGGAATCTGG |
|  |  |  |  |  | S2R: GGAGTCTCCCCTATTCCACG |
| SLCO1B3 | rs2117032 | 12 | 3’-UTR | HRM | S3F: TTGGCATATCATTAATTGTG |
|  |  |  |  |  | S3R: GCAAACCTTTAGCAAGACTA |
|  | rs2417940 | 12 | Intron 7 | HRM | S4F: ATGATGGTTAGAATGGGGAGA |
|  |  |  |  |  | S4R: TGGAAAACCAATCAGAAAGC |
| BLVRA | rs699512 | 7 | Exon 2 | HRM | B1F: GACCTGAACCTCTGCTTTTG |
|  |  |  |  |  | B1R: CATCCCTTAAACTGGTCTTTGTA |
