# Supplementary material for: Multiple Genetic Modifiers of Bilirubin Metabolism Involvement in Significant Neonatal Hyperbilirubinemia in Patients of Chinese Descent
Source: PLoS One. 2015 Jul 6;10(7):e0132034. doi: 10.1371/journal.pone.0132034 (PMC4493094; doi:10.1371/journal.pone.0132034)
Supplement: S2 Table — (DOC) [file pone.0132034.s002.doc]

Table S2 Linkage disequilibrium (LD) analysis of *UGT1A1* gene

|  | *rs4148323* | |  | *rs35390940* | |  | *rs6742078* | |  | *rs108124* | |
| --- | --- | --- | --- | --- | --- | --- | --- | --- | --- | --- | --- |
|  | D’ | r^2^ |  | D’ | r^2^ |  | D’ | r^2^ |  | D’ | r^2^ |
| (TA)_n_ | 0.998 | 0.03 |  | 0.825 | 0.104 |  | **0.999** | **0.885** |  | 0.996 | 0.03 |
| rs4148323 |  |  |  | 0.003 | - |  | 0.995 | 0.027 |  | 0.822 | 0.05 |
| rs35390940 |  |  |  |  |  |  | 0.828 | 0.117 |  | 0.747 | 0.003 |
| rs6742078 |  |  |  |  |  |  |  |  |  | 0.999 | 0.026 |
